# Expression of Growth Hormone in *Lactococcus lactis* and Its Effect on Intestinal Barrier Damage in Mice with DSS-Induced Colitis

**DOI:** 10.3390/ijms27209118

**Published:** 2026-10-14

**Authors:** Shujie Liu, Bo Deng, Yongming Li, Jie Wu, Ziwei Xu

**Affiliations:** Institute of Animal Husbandry and Veterinary Science, Zhejiang Academy of Agricultural Sciences, 298 Desheng Middle Road, Hangzhou 310021, China

**Keywords:** growth hormone, *Lactococcus lactis*, colitis, intestinal barrier repair, mucosal repair

## Abstract

Growth hormone has the potential to modulate inflammatory responses, alleviate intestinal injury, and preserve intestinal health. However, its limited availability from natural sources restricts further research and practical applications. Therefore, we optimized the coding sequence for porcine growth hormone (pGH) and constructed recombinant *Lactococcus lactis* (*L. lactis*) expressing pGH. The effect of *L. lactis* (pNZ8148-pGH) against intestinal damage was evaluated in mice with DSS-induced colitis. Orally administered *L. lactis* (pNZ8148-pGH) attenuated the symptoms of DSS-induced colitis, including weight loss, colon shortening, and elevated colonic damage scores (CDS), and improved colonic tissue morphology. Compared with the DSS group, *L. lactis* (pNZ8148-pGH) significantly reduced serum diamine oxidase (DAO) activity, colonic myeloperoxidase activity, and serum D-lactate and endotoxin levels while increasing colonic occludin and claudin-1 levels. Colonic tumor necrosis factor-α, interleukin-2 (IL-2), and interleukin-6 levels decreased significantly, whereas the interleukin-10 (IL-10) level increased after *L. lactis* (pNZ8148-pGH) administration. Furthermore, *L. lactis* (pNZ8148-pGH) significantly increased the numbers of colonic Ki67- and PCNA-positive cells and their positive indices. In addition, compared with the DSS group, *L. lactis* (pNZ8148) significantly decreased CDS, serum DAO activity, and colonic IL-2 level while increasing colonic occludin and IL-10 levels. In conclusion, oral administration of *L. lactis* (pNZ8148-pGH) maintained intestinal barrier integrity, suppressed inflammation, promoted mucosal repair, and alleviated DSS-induced colitis.

## 1. Introduction

Growth hormone (GH) is a single-chain polypeptide hormone composed of 191 amino acids with two disulfide bonds [1]. It is secreted primarily by the anterior pituitary gland and also by non-pituitary tissues, such as the prostate, breast, and colon. GH plays critical roles in regulating anabolic and catabolic processes, including postnatal growth, reproduction, longevity, and body homeostasis [2]. Furthermore, GH promotes protein synthesis and lipolysis, and regulates carbohydrate metabolism. Studies in animals and humans have shown that GH stimulates the activation, differentiation, and proliferation of various cell types, and promotes tissue growth and wound healing, including in the gastrointestinal system [3,4,5]. Through direct actions and indirect mechanisms mediated by the GH-IGF-I axis, GH exerts physiological effects by binding to its receptor (GHR) and activating downstream signaling and gene expression [6]. GH plays a beneficial role in preserving intestinal homeostasis and health.

GH is crucial for intestinal growth, regulation of inflammation, and intestinal homeostasis. The presence of GHR throughout the intestinal epithelium suggests that GH can act directly on the intestine [7]. Studies have shown that GH promotes the proliferation of intestinal epithelial and goblet cells, inhibits apoptosis, and facilitates the differentiation of intestinal stem cells toward Paneth and enteroendocrine cells [8,9]. In experimental models of colitis, sepsis, and inflammatory bowel disease (IBD), GH administration preserves intestinal structure, suppresses bacterial translocation, reinforces barrier function, and promotes intestinal tissue repair [10,11,12]. GH administration can also modulate cellular and humoral immunity, and enhance overall immune function. Conversely, GH deficiency leads to intestinal atrophy, primarily due to reduced mitotic activity in epithelial stem cells [13]. Although GH has critical physiological roles, studies examining the association between GH and intestinal homeostasis, particularly the potential of GH to prevent intestinal disorders, remain limited. Genetic engineering provides a cost-effective platform for the large-scale production of functional proteins in recombinant bacteria.

*Lactococcus lactis* (*L. lactis*), as a model lactic acid bacterium, has a long history of use in food fermentation and preservation, and is generally recognized as safe (GRAS) [14]. Unlike *Escherichia coli*, this strain has low intrinsic immunogenicity and does not produce endotoxins, lipopolysaccharides, or inclusion bodies [15]. Owing to its non-pathogenic, non-invasive, and food-grade properties, *L. lactis* has emerged as an attractive host for large-scale heterologous protein expression. In addition, *L. lactis* can tolerate gastric acid and bile, enabling its survival during transit through the gastrointestinal tract. Heterologous proteins produced in *L. lactis* are predominantly in a soluble form. When used as an oral delivery vehicle, recombinant *L. lactis* can be directly administered to animals by intragastric gavage, thereby avoiding complex purification of the target proteins. To date, *L. lactis* has been used to express various exogenous proteins, such as enzymes, bacterial and viral antigens, and bioactive peptides [16,17]. Recombinant *L. lactis* has been used as an oral delivery vehicle for therapeutic molecules, including pancreatitis-associated protein, cathelicidin-related antimicrobial peptide, *Mycobacterium leprae* Hsp65, and p62 protein, to combat gastrointestinal disorders [18,19,20,21].

GH plays a critical role in maintaining intestinal health and homeostasis; however, its limited availability from natural sources restricts further research and practical applications, particularly against colitis. As a food-grade lactic acid bacterium, *L. lactis* is an ideal candidate for the production and oral delivery of functional proteins to improve intestinal health. On this basis, we hypothesized that *L. lactis* expressing porcine growth hormone (pGH) could be successfully constructed and that oral administration of recombinant *L. lactis* would improve intestinal barrier function and protect against intestinal inflammation in animals. Therefore, in the present study, we constructed recombinant *L. lactis* (pNZ8148-pGH) using the nisin-controlled expression (NICE) system. The bioactivity of *L. lactis* (pNZ8148-pGH) was then evaluated in a dextran sulfate sodium (DSS)-induced colitis model. To our knowledge, this is the first study to use *L. lactis* expressing pGH and to investigate its therapeutic potential for protecting against intestinal damage.

## 2. Results

### 2.1. Detection of pGH Expression in L. lactis

The optimized pGH gene was cloned into pNZ8148 to generate pNZ8148-pGH, which was then transformed into *E. coli* MC1061. Subsequently, the recombinant plasmid extracted from this intermediate host was introduced into *L. lactis* NZ9000, as confirmed by sequence analysis. Whole-cell lysates of *L. lactis* (pNZ8148-pGH) and *L. lactis* (pNZ8148) were analyzed by SDS-PAGE (Figure 1A). The SDS-PAGE gel showed an additional band of approximately 22 kDa in the *L. lactis* (pNZ8148-pGH) lane, corresponding to the expected molecular weight of pGH, whereas no such band was observed in the control strain *L. lactis* (pNZ8148) (*L. lactis*). Densitometric analysis indicated that the recombinant protein constituted approximately 9.3% of the total soluble cellular protein. Western blot analysis further confirmed a specific pGH band in *L. lactis* (pNZ8148-pGH), which was absent from the control *L. lactis* (Figure 1B). These results indicated that pGH was successfully expressed in recombinant *L. lactis*.

### 2.2. L. lactis (pNZ8148-pGH) Attenuated the Symptoms of DSS-Induced Colitis

To investigate the protective effect of *L. lactis* (pNZ8148-pGH) against intestinal inflammation and mucosal injury, mice with DSS-induced colitis were orally administered recombinant *L. lactis* (pNZ8148-pGH) or *L. lactis* for 12 days (Figure 2A). Mice with DSS-induced colitis exhibited bloody stools, significant weight loss, and colon shortening compared with the control (CON) group (*p* < 0.05; Figure 2B,C). However, oral administration of *L. lactis* (pNZ8148-pGH) (LL-pGH) significantly increased body weight gain and colon length compared with the DSS and *L. lactis* (LL) groups (*p* < 0.05), and mice in the LL-pGH group did not develop bloody stools. Furthermore, DSS treatment led to significantly elevated liver and kidney indices, whereas administration of either *L. lactis* (pNZ8148-pGH) or *L. lactis* significantly reduced both indices (*p* < 0.05), with no significant differences among the LL-pGH, LL, and CON groups (*p* > 0.05; Figure 2D,E).

### 2.3. L. lactis (pNZ8148-pGH) Attenuated DSS-Induced Colonic Structural Damage

The morphology of colonic tissue was examined histologically (Figure 3A). The colons of healthy mice in the CON group exhibited an intact epithelium and normal tissue architecture; no ulcers or inflammatory cell infiltration were observed. However, DSS treatment disrupted normal colonic architecture, causing shedding of the mucosal epithelium and exposure of the submucosal and muscular layers, which are characteristic of mucosal injury and ulceration. Oral administration of *L. lactis* (pNZ8148-pGH) improved colonic histology, with less ulceration and inflammatory cell infiltration while preserving normal tissue architecture. Although *L. lactis* moderately improved colonic architecture, localized ulcers with inflammatory cell infiltration remained visible. Furthermore, DSS treatment resulted in significantly higher colonic damage scores (CDS) than those in the CON group (*p* < 0.05; Figure 3B). Compared with the DSS group, oral administration of *L. lactis* (pNZ8148-pGH) or *L. lactis* significantly decreased CDS (*p* < 0.05), and the LL-pGH group showed lower CDS than the LL group (*p* < 0.05).

### 2.4. L. lactis (pNZ8148-pGH) Improved Intestinal Permeability in DSS-Induced Colitis

Serum markers of intestinal permeability, including diamine oxidase (DAO), D-lactate (D-LA), and endotoxin (ET), were measured (Figure 4A–C). Compared with the CON group, DSS treatment significantly increased serum DAO activity and D-LA and ET levels (*p* < 0.05). In contrast, oral administration of *L. lactis* (pNZ8148-pGH) significantly attenuated the increases in all three parameters relative to the DSS group (*p* < 0.05), and no significant differences were observed between the CON and LL-pGH groups (*p* > 0.05). Additionally, the LL group showed significantly lower DAO activity than the DSS group but higher DAO activity than the CON and LL-pGH groups (*p* < 0.05). No significant differences were observed in serum D-LA and ET levels among the CON, LL, and LL-pGH groups (*p* > 0.05).

Tight junction proteins provide key structural support for maintaining intestinal barrier integrity. In colonic tissue, myeloperoxidase (MPO) activity and levels of the tight junction proteins occludin and claudin-1 were also measured (Figure 4D–F). Compared with the CON group, DSS treatment significantly increased colonic MPO activity while reducing occludin and claudin-1 levels (*p* < 0.05). Conversely, oral administration of *L. lactis* (pNZ8148-pGH) effectively attenuated these DSS-induced changes (*p* < 0.05) and restored these parameters to near-normal levels. Additionally, colonic occludin level in the LL group was significantly higher than that in the DSS group (*p* < 0.05) but remained lower than that in the CON and LL-pGH groups (*p* < 0.05). No significant differences were observed in MPO activity or claudin-1 level between the LL and DSS groups (*p* > 0.05).

### 2.5. L. lactis (pNZ8148-pGH) Attenuated DSS-Induced Colonic Inflammation

To explore the effect of *L. lactis* (pNZ8148-pGH) on colonic inflammatory responses, we measured key pro-inflammatory (TNF-α, IL-1β, IL-2, and IL-6) and anti-inflammatory (IL-10 and IL-4) cytokines (Figure 5A–F). Compared with the CON group, DSS treatment significantly increased colonic TNF-α, IL-1β, IL-2, and IL-6 levels (*p* < 0.05) and significantly decreased IL-10 and IL-4 levels (*p* < 0.05). Compared with the DSS group, oral administration of *L. lactis* (pNZ8148-pGH) significantly reduced colonic TNF-α, IL-2, and IL-6 levels and increased the IL-10 level (*p* < 0.05). Except for IL-2, the levels of the other cytokines in the LL-pGH group did not differ significantly from those in the CON group (*p* > 0.05). In addition, *L. lactis* significantly reduced colonic IL-2 level and increased IL-10 level compared with the DSS group (*p* < 0.05), but IL-10 level remained lower than that in the LL-pGH group (*p* < 0.05).

### 2.6. L. lactis (pNZ8148-pGH) Promoted Proliferation of Colonic Epithelial Cells

The proliferation of colonic epithelial cells was evaluated by immunohistochemical staining for Ki67 and PCNA (Figure 6). Ki67- and PCNA-positive cells were predominantly distributed in the colonic mucosal epithelium and glandular regions. In these regions, the DSS group exhibited extensive inflammatory cell infiltration and fewer Ki67- and PCNA-positive cells. Moreover, the positive indices of both markers in the DSS group were significantly lower than those in the CON group. Oral administration of *L. lactis* (pNZ8148-pGH) increased the numbers of Ki67- and PCNA-positive cells and yielded significantly higher positive indices than those in the DSS and LL groups (*p* < 0.05), although the indices remained lower than those in the CON group. In addition, administration of *L. lactis* increased the numbers of both positive cell types compared with the DSS group; however, their positive indices did not differ significantly from those of the DSS group (*p* > 0.05).

**Figure 6:**
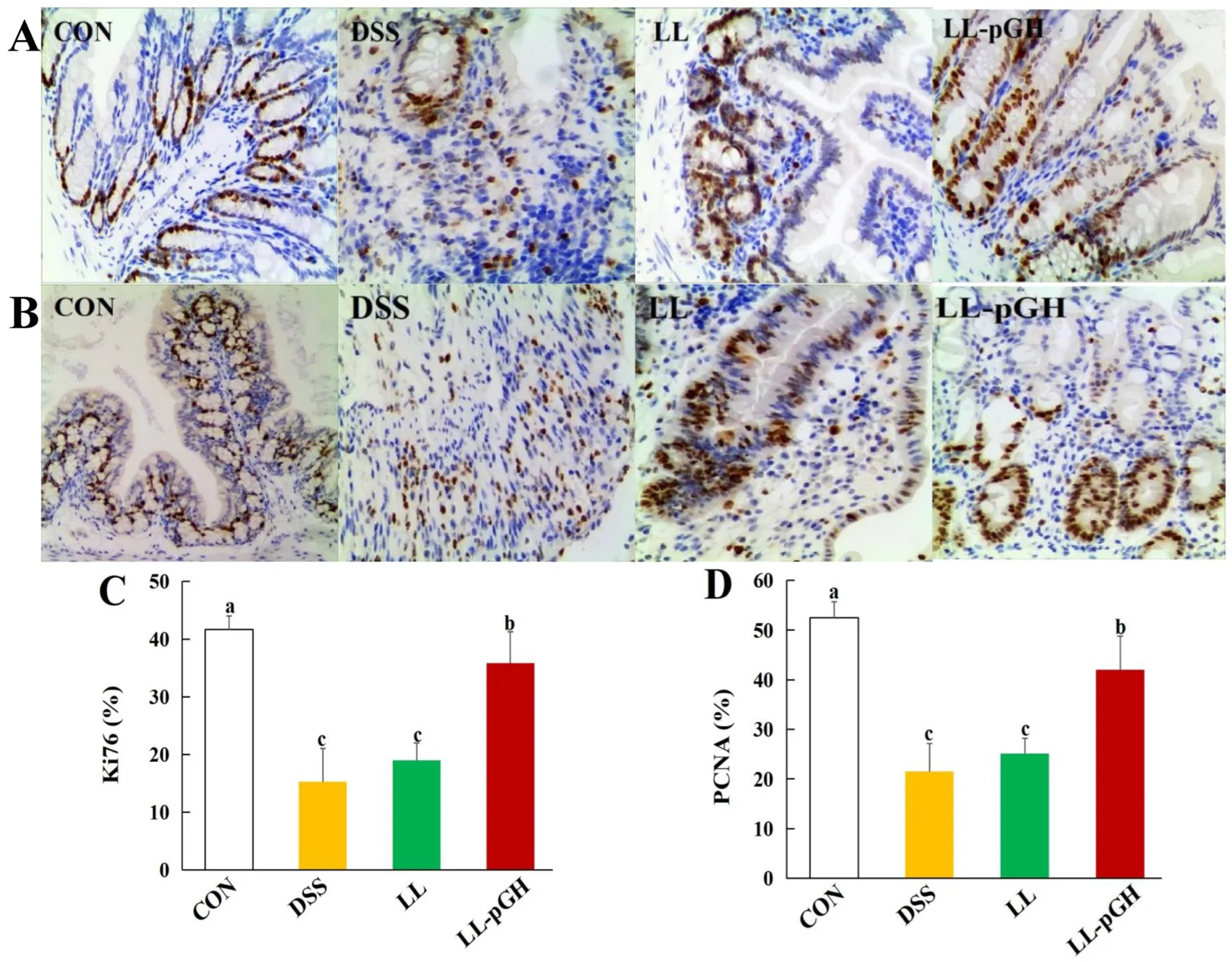
Effect of *L. lactis* (pNZ8148-pGH) on colonic epithelial cell proliferation in DSS-induced colitis. (**A**) Immunohistochemical staining for Ki67, Scale bar = 100 μm; (**B**) immunohistochemical staining for PCNA; (**C**) Ki67 index; (**D**) PCNA index. n = 10 mice per group. Data are expressed as the mean ± SD. Bars without a common letter differ significantly (*p* < 0.05). CON: normal control; DSS: DSS model control; LL: *L. lactis* (pNZ8148); LL-pGH: *L. lactis* (pNZ8148-pGH).

## 3. Discussion

GH is a pleiotropic hormone produced by the anterior pituitary gland and plays an important role in maintaining intestinal health and homeostasis. It stimulates the proliferation of intestinal epithelial cells and the differentiation of intestinal stem cells while inhibiting apoptosis of colonic epithelial cells [9,12]. Although GH has important biological functions, its limited availability and high cost of extraction from natural sources have hindered further research and practical applications in various fields. As a food-grade lactic acid bacterium, *L. lactis* has multiple probiotic functions, including improving intestinal microecology, modulating immune responses, and enhancing nutrient utilization efficiency in the host [22,23]. Given these advantages, it serves as an effective vector for producing and orally delivering functional proteins (e.g., largemouth bass ranavirus antigen, Gallus epidermal growth factor, and lipocalin 2) to combat intestinal diseases and improve gut health [24,25,26]. In the present study, recombinant *L. lactis* was successfully engineered to express pGH after codon optimization and vector construction. To our knowledge, this is the first study to use *L. lactis* as a delivery vector for pGH expression and to investigate the therapeutic potential of *L. lactis* (pNZ8148-pGH) for repairing intestinal damage in a DSS-induced colitis model.

The biological effects of *L. lactis* (pNZ8148-pGH) in this study were evaluated in a DSS-induced colitis model. As a chemical toxin, DSS directly injures colonic epithelial cells, causing epithelial erosion and ulceration. This disruption increases mucosal permeability and allows bacterial translocation into the mucosa. Subsequently, translocated bacteria trigger immune-cell infiltration and pro-inflammatory cytokine secretion, which ultimately propagate inflammation to deeper tissues [27]. In the present study, DSS treatment caused significant weight loss and colon shortening, disrupted colonic architecture, and elicited inflammatory-cell infiltration and mucosal damage, accompanied by an increased CDS. In contrast, oral administration of *L. lactis* (pNZ8148-pGH) effectively attenuated these manifestations of DSS-induced colitis, suppressed inflammatory-cell infiltration, mitigated histopathological damage, and restored epithelial architecture. Previous studies have shown that GH increases body weight and regulates tissue growth, while maintaining intestinal function and mucosal barrier integrity under stress conditions [28,29]. However, few studies have investigated the effects of GH on colitis. In patients with short bowel syndrome, low-dose recombinant human GH (rhGH) treatment increased body weight, lean mass, and fat-free mass, possibly owing to expansion of the intestinal surface area [30]. Compared with the non-transgenic group, GH-transgenic zebrafish showed significant increases in intestinal and whole-body mass and length, with an elevated enterosomatic index [31]. In a rat model of colonic anastomosis, GH increased body weight and anastomotic collagen deposition, reduced the incidence of rupture and dehiscence, promoted colonic healing, and mitigated the detrimental effects of intraperitoneally administered chemotherapy [32]. In rat models of TNBS-induced colitis or sepsis, GH reduced the extent of colonic ulcers, submucosal edema, and polymorphonuclear neutrophil infiltration and attenuated intestinal mucosal injury [10,33]. Consistent with these reports, the current study verified that *L. lactis* (pNZ8148-pGH) exhibited bioactivity related to intestinal homeostasis. Increasing evidence has revealed that the biological actions of GH on target cells are modulated by direct effects or indirect regulation mediated by IGF-I, a key intestinal growth factor synthesized locally or in the liver [33]. However, several studies have reported no beneficial effects of GH alone or combined with glutamine on intestinal structure and function [34]. Such inconsistent findings may be attributed to variations in GH dosage, formulation, treatment duration, co-administered nutritional supplements, and intestinal disease types. In addition, studies have demonstrated that *L. lactis* can improve intestinal architecture and alleviate intestinal injury in various intestinal diseases. Oral administration of *L. lactis* NK209 suppressed colon shortening in mice with TNBS-induced colitis [35]. In mice with DSS-induced colitis, administration of heat-killed *L. lactis* BF3 attenuated colon shortening and mucosal damage [36]. Alves et al. reported that *L. lactis* did not prevent weight loss and colon shortening, but it improved the global macroscopic disease index, mitigated goblet cell depletion, and alleviated colonic histological damage in DSS-induced colitis [37]. Similarly, our results showed that oral administration of *L. lactis* improved CDS and maintained normal colonic architecture. These results suggest that, in addition to serving as a delivery vehicle, *L. lactis* also exerts beneficial effects against DSS-induced colitis to some extent. Taken together, oral administration of *L. lactis* (pNZ8148-pGH) effectively alleviated symptoms of DSS-induced colitis in mice, preserved colonic architecture, and mitigated colonic damage.

The intestine is a vital organ for nutrient digestion and absorption and a major immune organ. As the primary line of defense, an intact intestinal barrier is essential for maintaining homeostasis and health. However, intestinal barrier impairment increases epithelial permeability, facilitates the translocation of toxins and pathogens into host tissues, and triggers inflammatory responses [38]. Tight junctions (TJs), which consist mainly of occludin and claudin proteins, are key structural components of the intestinal epithelial barrier [39]. These structures seal the paracellular space and are critical determinants of paracellular permeability and epithelial barrier integrity [40]. Serum levels of DAO, D-LA, and ET are widely used as biochemical markers for assessing intestinal permeability and indirectly reflect the severity of intestinal barrier damage [41]. When the barrier is compromised and permeability increases, intestinal occludin and claudin levels decline, whereas DAO, D-LA, and ET are released into the circulation [41,42]. Our study revealed that 3.5% DSS markedly elevated serum DAO activity and ET and D-LA levels while reducing colonic claudin-1 and occludin levels in mice, indicating compromised barrier integrity and increased permeability. However, oral administration of *L. lactis* (pNZ8148-pGH) reduced the levels of these serum markers and increased the levels of colonic TJ proteins. These findings suggest that *L. lactis* (pNZ8148-pGH) mitigates DSS-induced intestinal hyperpermeability and preserves barrier integrity. Studies on the effects of GH on intestinal permeability remain limited. Wang et al. showed that GH administration markedly lowered plasma ET levels, serum amylase and lipase activities, and the incidence of bacterial translocation, and alleviated intestinal hyperpermeability in rats with acute necrotizing pancreatitis [33]. In scalded rats, rhGH combined with enteral immunonutrition reduced serum ET and TNF-α levels, and reinforced intestinal mucosal barrier protection [43]. However, chronic GH elevation altered intestinal epithelial intercellular architecture and elevated paracellular permeability, which likely represent an adaptive mechanism to accommodate the elevated energy demands of anabolic metabolism [31]. Furthermore, some studies have shown that *L. lactis* can enhance intestinal barrier integrity by upregulating TJ protein expression. Supplementation with *L. lactis* ZB2 significantly decreased serum permeability markers (FITC-dextran flux, LPS, DAO, and D-LA), upregulated the expression of TJ proteins (ZO-1, claudin-1, and occludin), and improved intestinal barrier sealing function and epithelial integrity [44]. In animal models of colitis or cirrhosis, administration of *L. lactis* HF08 or NZ9000 effectively modulated intestinal paracellular permeability, suppressed bacterial translocation and preserved epithelial barrier integrity [45,46]. Our results suggest that *L. lactis* also moderately restored intestinal integrity, as evidenced by an increase in colonic occludin level and a decrease in serum DAO activity, whereas oral administration of *L. lactis* (pNZ8148-pGH) repaired intestinal damage and more effectively maintained barrier integrity, indirectly suggesting the dual effects of *L. lactis* and rpGH.

Intestinal inflammation triggered by an imbalance between pro- and anti-inflammatory cytokines plays a crucial role in the pathogenesis of diverse intestinal diseases. Overproduction of pro-inflammatory cytokines (e.g., TNF-α, IL-1β, IL-2, and IL-6) disrupts intestinal homeostasis, increases permeability, and drives sustained inflammation and intestinal damage [47]. As a pivotal inflammatory regulator, TNF-α modulates acute and chronic inflammatory responses and plays a central role in the pathogenesis of autoimmune conditions such as ulcerative colitis [48]. IL-1β stimulates the secretion of diverse inflammatory mediators, thereby initiating and amplifying inflammatory reactions [49]. IL-2 is critical for regulatory T-cell homeostasis, and its dysregulation contributes to intestinal disease pathogenesis [50]. Under inflammatory conditions, IL-6, a pleiotropic pro-inflammatory cytokine, disrupts the intestinal epithelial barrier by increasing intestinal permeability. As a broadly inhibitory cytokine, IL-10 suppresses pro-inflammatory responses and modulates adaptive immune responses triggered by tissue damage [51]. IL-4 regulates immune responses and drives tissue repair through complex signaling cascades [52]. In addition, MPO, which is primarily stored in neutrophils, is released extracellularly during inflammation and is widely recognized as a valuable marker of inflammatory severity [53]. Our results showed that DSS significantly increased colonic MPO activity and TNF-α, IL-1β, IL-2, and IL-6 levels while decreasing IL-10 and IL-4 levels, indicating neutrophil infiltration, aberrant immune responses, and colonic inflammation. Conversely, oral administration of *L. lactis* (pNZ8148-pGH) markedly limited neutrophil infiltration, suppressed pro-inflammatory cytokine release, and restored the anti-inflammatory IL-10 level, thereby ameliorating DSS-induced colitis. This protection likely stems from the ability of *L. lactis* (pNZ8148-pGH) to preserve intestinal barrier integrity and limit bacterial translocation. Studies have indicated that GH participates in immune system development and enhances overall immune function, and that its receptor is expressed on several leukocyte subpopulations [54]. Glutamine and rhGH administration enhanced immune function and modulated the inflammatory response in patients after portal hypertension surgery, with significant increases in CD3, CD4/CD8, IgG, and IL-2 levels and decreases in CRP and TNF-α levels [54]. In rats subjected to partial intestinal obstruction, rhGH treatment elevated IgA concentration and alleviated intestinal mucosal injury and subsequent bacterial translocation [55]. Co-administration of EN and rhGH significantly increased intestinal sIgA level and the number of IgA plasma cells, reduced serum ET and TNF-α concentrations, and strengthened mucosal barrier protection in scalded rats with intestinal damage [43]. In addition, previous studies have demonstrated that *L. lactis* has anti-inflammatory properties and immunomodulatory effects in intestinal diseases. In DSS-induced colitis models, *L. lactis* NZ9000 or HF08 suppressed intestinal inflammation and alleviated immune dysfunction by reducing pro-inflammatory cytokines (TNF-α, IL-6, and IL-1β) and increasing IL-10 in the colon [45,46]. *L. lactis* ZB2 modulated immune homeostasis toward an anti-inflammatory phenotype by down-regulating colonic pro-inflammatory cytokines, up-regulating Reg3γ and β-defensin 1, and increasing serum IL-10 and TGF-β levels [44]. Similarly, our results indicated that *L. lactis* significantly lowered colonic IL-2 level while elevating IL-10 level compared with DSS-treated mice. These findings suggest that *L. lactis* has anti-inflammatory properties and preserves intestinal barrier integrity to a certain extent, supporting its potential as a promising expression and delivery vector. Oral administration of *L. lactis* (pNZ8148-pGH) alleviated intestinal inflammatory responses and exerted anti-inflammatory and mucosal protective effects in mice with DSS-induced colitis.

Cell proliferation is a core driver of tissue regeneration, and the efficiency and integrity of tissue repair depend heavily on cellular regenerative potential and proliferative activity. Ki-67, a nuclear protein encoded by the *MKI67* gene, is expressed in proliferating mammalian cells. It is absent in quiescent (G0) cells, appears in the G1, S, and G2 phases, and peaks in M phase [56]. Because of its strong association with cell growth and division, Ki-67 is considered a reliable marker of cellular proliferation [57]. PCNA is a nuclear protein that accumulates in the late G1 and early S phases and is involved in cell-cycle progression, DNA replication, and repair, making it a useful marker of cellular proliferation [58]. Studies have shown that DSS treatment reduces the number of proliferating colonic cells and increases the quiescent (G0) cell population, leading to cell-cycle arrest rather than proliferation [59]. In the present study, DSS markedly decreased the numbers and indices of Ki67- and PCNA-positive cells in colonic tissues, indicating fewer proliferating colonic epithelial cells than in the CON group. In addition, the majority of PCNA-positive nuclei belonged to stromal cells in the DSS group, which was most likely attributable to the activation of inflammatory cells in the lamina propria. This was consistent with the findings that DSS disrupts colonic tissue architecture and triggers inflammatory responses. Conversely, oral administration of *L. lactis* (pNZ8148-pGH) significantly elevated the numbers and indices of Ki67- and PCNA-positive cells, suggesting that it promoted regeneration of the damaged epithelium. Ersoy et al. demonstrated that GH administration stimulated proliferation of intestinal mucosal goblet cells, crypt epithelial cells, and enteroendocrine cells in rats [8]. In experimental models of colonic anastomosis, short bowel syndrome (SBS), and scald-induced intestinal injury, GH administration significantly increased the number of PCNA-positive cells and the PCNA value or index in intestinal tissues. GH also stimulated enterocyte proliferation and facilitated intestinal wound healing [43,55,60]. Consistent with these findings, the results of this study indicated that oral administration of *L. lactis* (pNZ8148-pGH) promoted colonic epithelial cell proliferation, accelerated recovery from colonic injury, and attenuated DSS-induced colitis. However, *L. lactis* did not significantly affect the indices of Ki67- and PCNA-positive cells in mice with DSS-induced colitis.

In the present study, *L. lactis* itself exerted moderate protective effects against DSS-induced colitis, supporting its suitability as an expression and oral delivery vector. However, due to the lack of a purified rpGH treatment group, we could not accurately quantify the extent of intestinal protection attributable to rpGH or the *L. lactis* carrier in *L. lactis* (pNZ8148-pGH), or determine whether administration of this recombinant strain was superior to conventional GH protein alone. In addition, we used an acute DSS-induced colitis model to evaluate the protective effect of *L. lactis* (pNZ8148-pGH) against intestinal injury; however, its long-term efficacy and safety were not evaluated. Furthermore, the release dynamics, biological activity, and intestinal translocation of rpGH in vivo were not characterized following oral administration of recombinant *L. lactis*. These aspects should be examined in future studies. In addition, the exact molecular mechanisms and signaling pathways through which *L. lactis* (pNZ8148-pGH) promotes intestinal repair and preserves barrier integrity require further investigation.

In conclusion, *L. lactis* expressing porcine growth hormone (pGH) was successfully constructed. Our study demonstrated that oral administration of *L. lactis* (pNZ8148-pGH) effectively alleviated the symptoms of DSS-induced colitis in mice, preserved normal colonic architecture, reduced intestinal permeability, suppressed the inflammatory response, promoted epithelial cell proliferation, and facilitated colonic tissue repair. These findings provide new insights and an experimental foundation for using *L. lactis* (pNZ8148-pGH) to protect against intestinal inflammation and damage.

## 4. Materials and Methods

### 4.1. Bacterial Strains and Growth Conditions

The host strain *L. lactis* NZ9000 and plasmid pNZ8148 (an intracellular expression vector) from the NICE system were kindly provided by NIZO Food Research. *L. lactis* NZ9000 was cultivated in M17 medium (Difco, Sparks, MD, USA) containing 0.5% glucose (GM17) at 30 °C without shaking. For recombinant *L. lactis* carrying the plasmid, the growth medium was supplemented with 10 μg/mL chloramphenicol. *Escherichia coli* (*E. coli*) MC1061, which served as the intermediate cloning host, was kindly provided by Professor Weifen Li at Zhejiang University, Hangzhou, China. This strain was grown in Tryptone Soya broth or Luria-Bertani medium at 37 °C.

### 4.2. Optimization of the pGH Gene Fragment

To enhance the yield of rpGH in *L. lactis*, the truncated pGH gene lacking its signal sequence was designed and optimized based on *L. lactis* codon preference, with low-usage codons substituted with high-usage counterparts. An NcoI restriction site containing a start codon (CCATGG) was incorporated at the 5′ end of the pGH gene, while a stop codon followed by a HindIII restriction site (AAGCTT) was added to the 3′ end of the gene. Additionally, the G+C content was adjusted to within the range of 40% to 70%. The designed gene sequence was synthesized by Sangon Biotech Co., Ltd. (Shanghai, China). After synthesis, the gene fragment was sequenced to confirm that the correct gene fragment had been obtained.

### 4.3. Construction of Recombinant L. lactis Expressing pGH

The synthesized gene fragment (pGH) was inserted into the NcoI and HindIII sites of the pNZ8148 expression vector. The recombinant plasmid was then transformed into *E. coli* MC1061. After verification by DNA sequencing, the plasmid designated pNZ8148-pGH was transformed into *L. lactis* NZ9000 by electroporation. Recombinant *L. lactis* strains were selected using 10 μg/mL chloramphenicol, and positive clones were verified by NcoI and HindIII digestion and subsequent sequencing (Sangon Biotech Co., Ltd., Shanghai, China). Recombinant *L. lactis* harboring pNZ8148-pGH was designated *L. lactis* (pNZ8148-pGH).

### 4.4. Expression and Identification of pGH in L. lactis

Recombinant pGH expressed in *L. lactis* was detected by sodium dodecyl sulfate-polyacrylamide gel electrophoresis (SDS-PAGE) and Western blotting. *L. lactis* (pNZ8148-pGH) and *L. lactis* (pNZ8148) were cultivated in GM17 medium. When the strains reached an optical density (OD600) of 0.5, nisin (Sigma-Aldrich, St. Louis, MO, USA) was added to the medium at a final concentration of 10 ng/mL, and the cultures were incubated for 4 h to induce rpGH production. After induction, the strains were harvested by centrifugation at 3000× *g* for 15 min. Cell extracts were prepared by mechanical disruption with glass beads (0.15–0.25 mm diameter; Sigma, St. Louis, MO, USA) using a MiniBeadBeater. Aliquots (10 μL) of the cell extracts were loaded onto SDS-PAGE, and the recombinant protein in the gels was quantified using Quantity One software version 4.62 (Bio-Rad, CA, USA). The percentage of the target protein was calculated as the ratio of the target band’s grayscale value to the total grayscale values of all bands in the same lane, and assays were performed in triplicate. Proteins separated by SDS-PAGE were subsequently transferred to a polyvinylidene fluoride membrane (Millipore, Bedford, MA, USA). After blocking with 5% BSA (*w/v*) in phosphate-buffered saline (PBS) for 2 h at room temperature, the membrane was incubated with an anti-GH polyclonal antibody (LifeSpan BioSciences, Seattle, WA, USA) overnight at 4 °C, and then with an HRP-conjugated anti-rabbit IgG secondary antibody (Abcam, Cambridge, MA, USA) for 2 h at room temperature. Finally, protein bands were visualized using 3,3′-diaminobenzidine tetrahydrochloride (DAB; Sangon Biotech, Shanghai, China) as the chromogenic substrate.

### 4.5. Preparation of the Strains for Animal Experiments

*L. lactis* (pNZ8148-pGH) and *L. lactis* (pNZ8148) were cultured and induced as described above. The strains were harvested by centrifugation at 3000× *g* for 15 min, followed by three washes with sterile PBS. Finally, the pellets were resuspended in PBS to a density of 2 × 10^12^ CFU per 0.2 mL for oral administration.

### 4.6. Induction of Colitis by Dextran Sulfate Sodium

To evaluate the potential of *L. lactis* (pNZ8148-pGH) to protect against intestinal inflammation and injury, ulcerative colitis was induced in mice using dextran sulfate sodium (DSS; MP Biomedicals, Santa Ana, CA, USA). Female BALB/c mice (8 weeks old, specific-pathogen-free) were purchased from SLAC Laboratory Animal Center (Shanghai, China). The mice were housed in an experimental animal room under a 12-h light/dark cycle at a temperature of 25 ± 1 °C, with free access to food and water. Following a 7-day acclimation period, the mice were randomly allocated into four groups (n = 10 per group): normal control (CON), DSS model control (DSS), *L. lactis* (pNZ8148) (LL), and *L. lactis* (pNZ8148-pGH) (LL-pGH). The CON group received normal drinking water for 12 days. All other groups received 3.5% (*w/v*) DSS solution (36,000–50,000 MW; MP Biomedicals, LLC, Illkirch, France) for 7 days, followed by normal drinking water for 5 days. In addition, from day 1 to day 12, mice in the LL and LL-pGH groups were orally administered 2 × 10^12^ CFU of *L. lactis* (pNZ8148) or *L. lactis* (pNZ8148-pGH), respectively, while mice in the CON and DSS groups received an equivalent volume of PBS daily. Body weight was recorded throughout the experimental period. On day 13, blood samples were obtained from all mice via orbital puncture, and serum was isolated by centrifugation at 3000× *g* for 15 min at 4 °C. Subsequently, all mice were anesthetized with pentobarbital sodium and euthanized. The length of the colon was measured, and colonic tissues were collected for further analysis.

### 4.7. Histological Analysis and Damage Score of Colitis

Following standard histological procedures, distal colon tissue samples of all mice (approximately 1 cm) were embedded in paraffin wax and cut into 4 μm sections. The sections were stained with hematoxylin and eosin (H&E), and five slide images from each mouse were independently examined under a light microscope. As described by Dieleman et al., histological damage of the colon was graded in a blinded manner [61]. Briefly, the severity of colonic inflammation (acute or chronic) was scored on a scale from 0 to 3. The depth of inflammation and the degree of crypt damage or regeneration were scored on a scale from 0 to 4. These results were further graded based on the percentage of involvement in the disease process: (1) 1–25%, (2) 26–50%, (3) 51–75%, and (4) 76–100%. Each colonic section was scored for each feature by multiplying the grade by its percentage involvement. Finally, the colonic damage score (CDS) of each section was calculated as the sum of these individual scores, and the CDS of each mouse was calculated as the mean of five slide images per colon.

### 4.8. Enzyme-Linked Immunosorbent Assay (ELISA)

Colon tissue samples were homogenized in RIPA lysis buffer containing phosphatase inhibitors. The homogenate was centrifuged at 3000× *g* for 15 min at 4 °C, and the supernatant was harvested. Total protein concentration was measured using a BCA protein assay kit. Finally, cytokine concentrations (TNF-α, IL-1β, IL-2, IL-6, IL-10, and IL-4) were measured using the corresponding ELISA kits according to the manufacturer’s instructions (Nanjing Jiancheng Bioengineering Co., Ltd., Nanjing, China).

### 4.9. Assessment of Colon Permeability

Colonic MPO activity was measured using an assay kit according to the manufacturer’s instructions (Jiancheng Bio, Nanjing, China). Colonic occludin and claudin-1 levels were detected using ELISA kits (Jiancheng Bio, Nanjing, China). Additionally, serum DAO activity and ET and D-LA levels were measured using corresponding commercial kits (Jiancheng Bio, Nanjing, China).

### 4.10. Immunohistochemistry

Immunohistochemistry for Ki-67 and PCNA was performed on distal colon sections from all mice. Briefly, paraffin-embedded colon sections were deparaffinized using xylene and rehydrated through a graded alcohol series. Antigen retrieval was performed using citrate buffer, followed by blocking of endogenous peroxidase with 3% H_2_O_2_ for 15 min. Sections were incubated with anti-Ki-67 antibody or anti-PCNA antibody overnight at 4 °C, followed by three PBS washes. Afterward, sections were incubated with the corresponding secondary antibodies at 37 °C for 30 min. The sections were developed with DAB and counterstained with hematoxylin; five slide images from each mouse were examined under light microscopy. For quantitative analysis, positively stained cells were counted among more than 200 cells at 200× magnification. The positivity index was subsequently calculated as the percentage of positive cells relative to the total number of cells counted. Finally, the positively stained cell numbers and positive indices for each mouse were the mean of five slide images per colon.

### 4.11. Statistical Analysis

All experimental data were analyzed using SPSS version 21.0 (SPSS, Inc., Chicago, IL, USA). Data are presented as the mean ± standard deviation (SD). Statistical differences between two groups were evaluated using Student’s *t*-test, whereas comparisons among multiple groups were performed using one-way ANOVA with Tukey’s multiple-comparison test. A value of *p* < 0.05 was considered statistically significant.

## Figures and Tables

**Figure 1:**
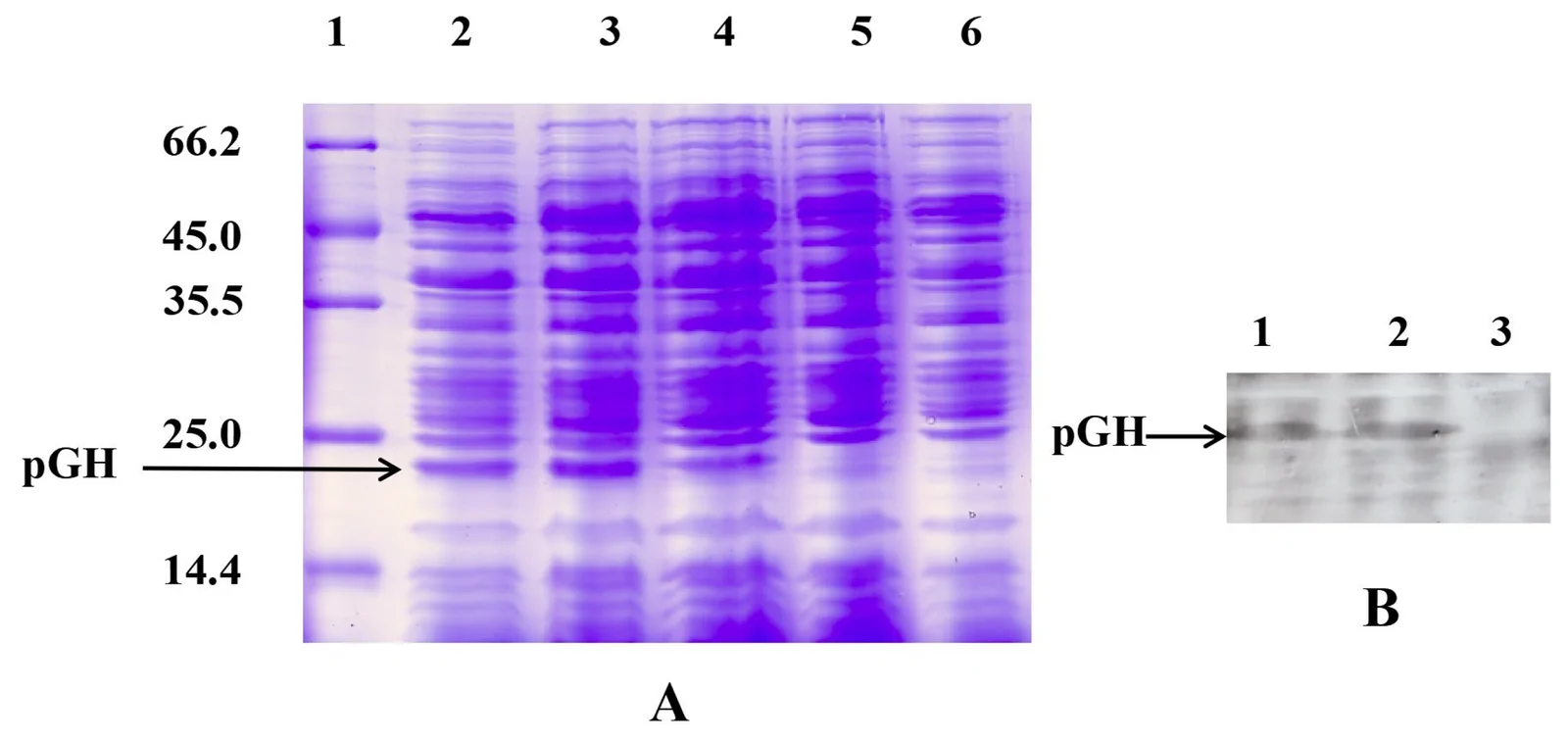
Detection of pGH expression in *L. lactis* by SDS-PAGE and Western blotting. (**A**) Detection of pGH by SDS-PAGE; lane 1, protein marker; lanes 2–4, *L. lactis* (pNZ8148-pGH); lanes 5 and 6, *L. lactis* (pNZ8148). (**B**) Detection of pGH by Western blotting; lanes 1 and 2, *L. lactis* (pNZ8148-pGH); lane 3, *L. lactis* (pNZ8148).

**Figure 2:**
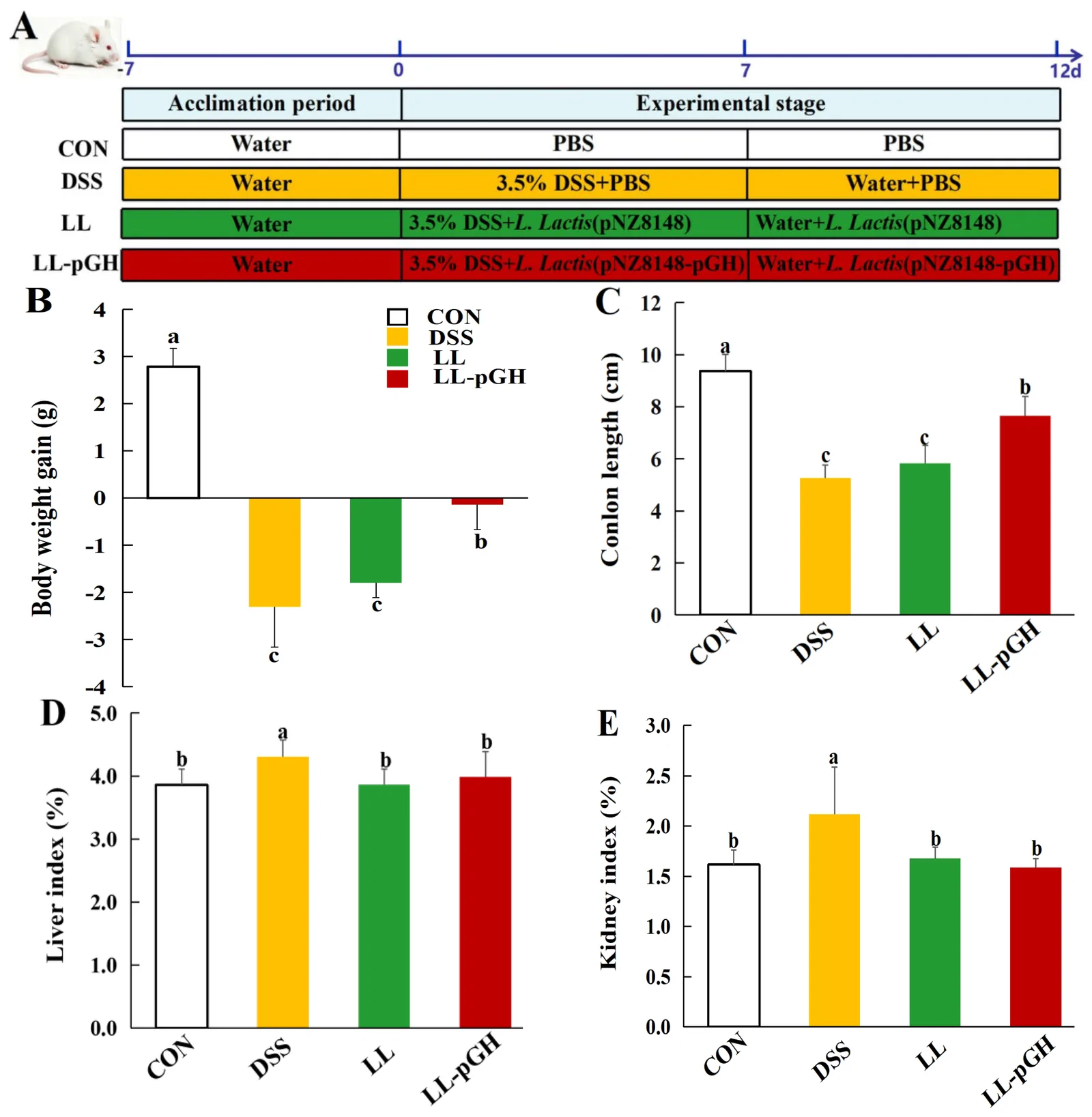
*L. lactis* (pNZ8148-pGH) alleviated the pathological symptoms of DSS-induced colitis. (**A**) Experimental design of the animal trial; (**B**) body weight gain during days 0–12; (**C**) colon length; (**D**) liver index; (**E**) kidney index. n = 10 mice per group. Data are expressed as the mean ± SD. Bars without a common letter differ significantly (*p* < 0.05). CON: normal control; DSS: DSS model control; LL: *L. lactis* (pNZ8148); LL-pGH: *L. lactis* (pNZ8148-pGH).

**Figure 3:**
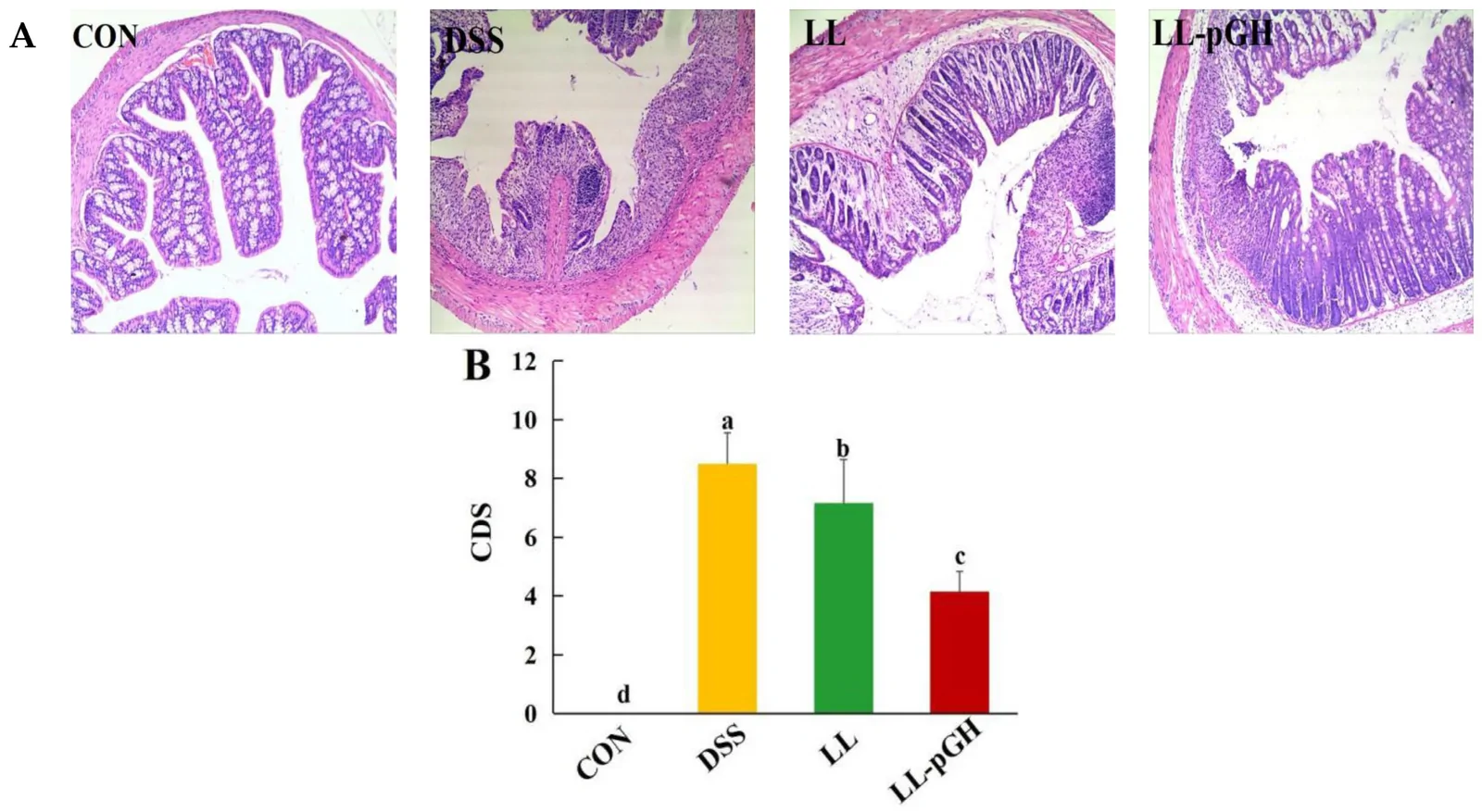
*L. lactis* (pNZ8148-pGH) ameliorated intestinal barrier damage in DSS-induced colitis. (**A**) Representative H&E staining of colonic tissues, Scale bar = 500 μm; (**B**) colonic damage score (CDS). n = 10 mice per group. Data are expressed as the mean ± SD. Bars without a common letter differ significantly (*p* < 0.05). CON: normal control; DSS: DSS model control; LL: *L. lactis* (pNZ8148); LL-pGH: *L. lactis* (pNZ8148-pGH).

**Figure 4:**
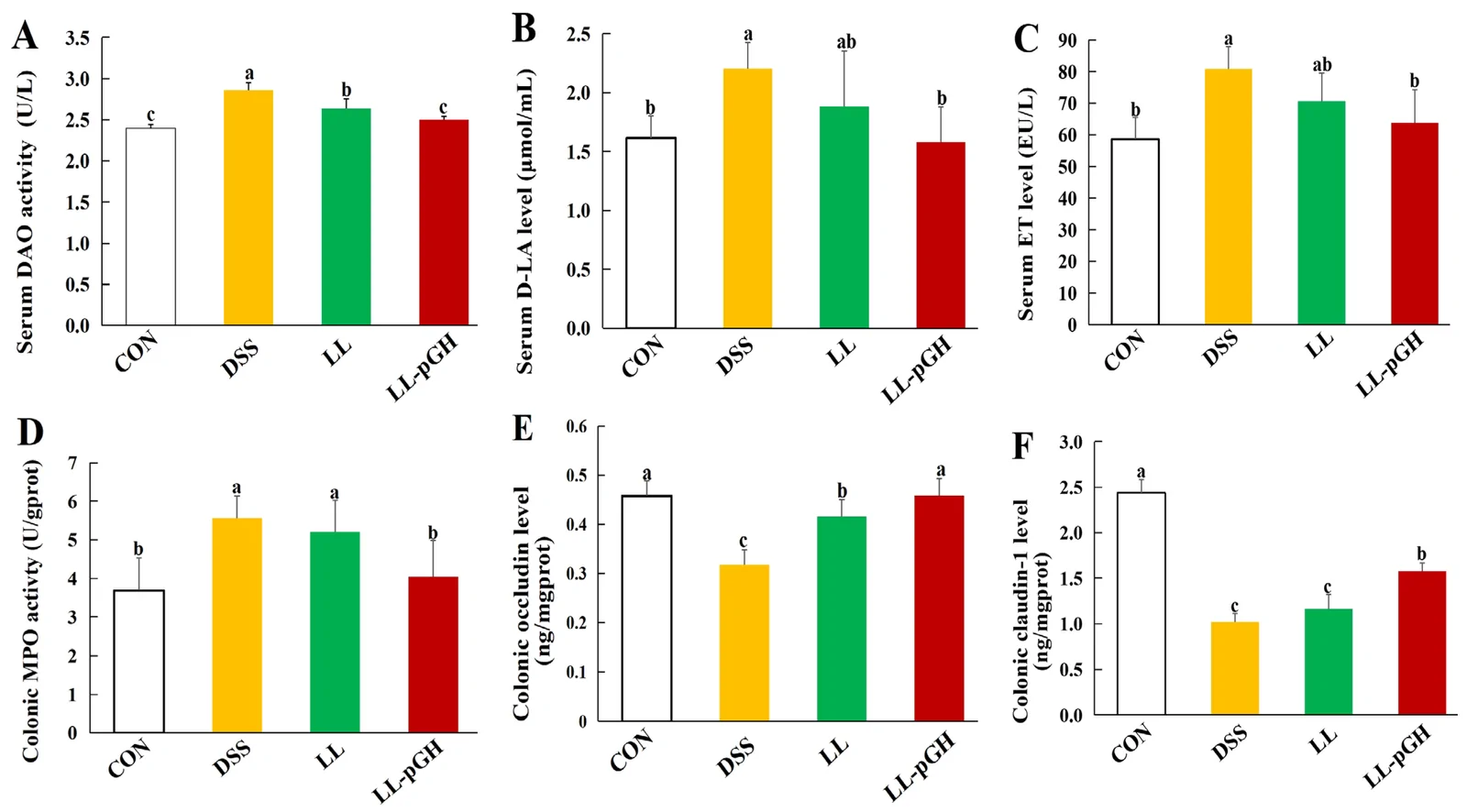
Effect of *L. lactis* (pNZ8148-pGH) on intestinal permeability in DSS-induced colitis. (**A**) Serum DAO activity; (**B**) serum D-LA level; (**C**) serum ET level; (**D**) colonic MPO activity; (**E**) colonic occludin level; (**F**) colonic claudin-1 level. MPO, myeloperoxidase; DAO, diamine oxidase; ET, endotoxin. n = 10 mice per group. Data are expressed as the mean ± SD. Bars without a common letter differ significantly (*p* < 0.05). CON: normal control; DSS: DSS model control; LL: *L. lactis* (pNZ8148); LL-pGH: *L. lactis* (pNZ8148-pGH).

**Figure 5:**
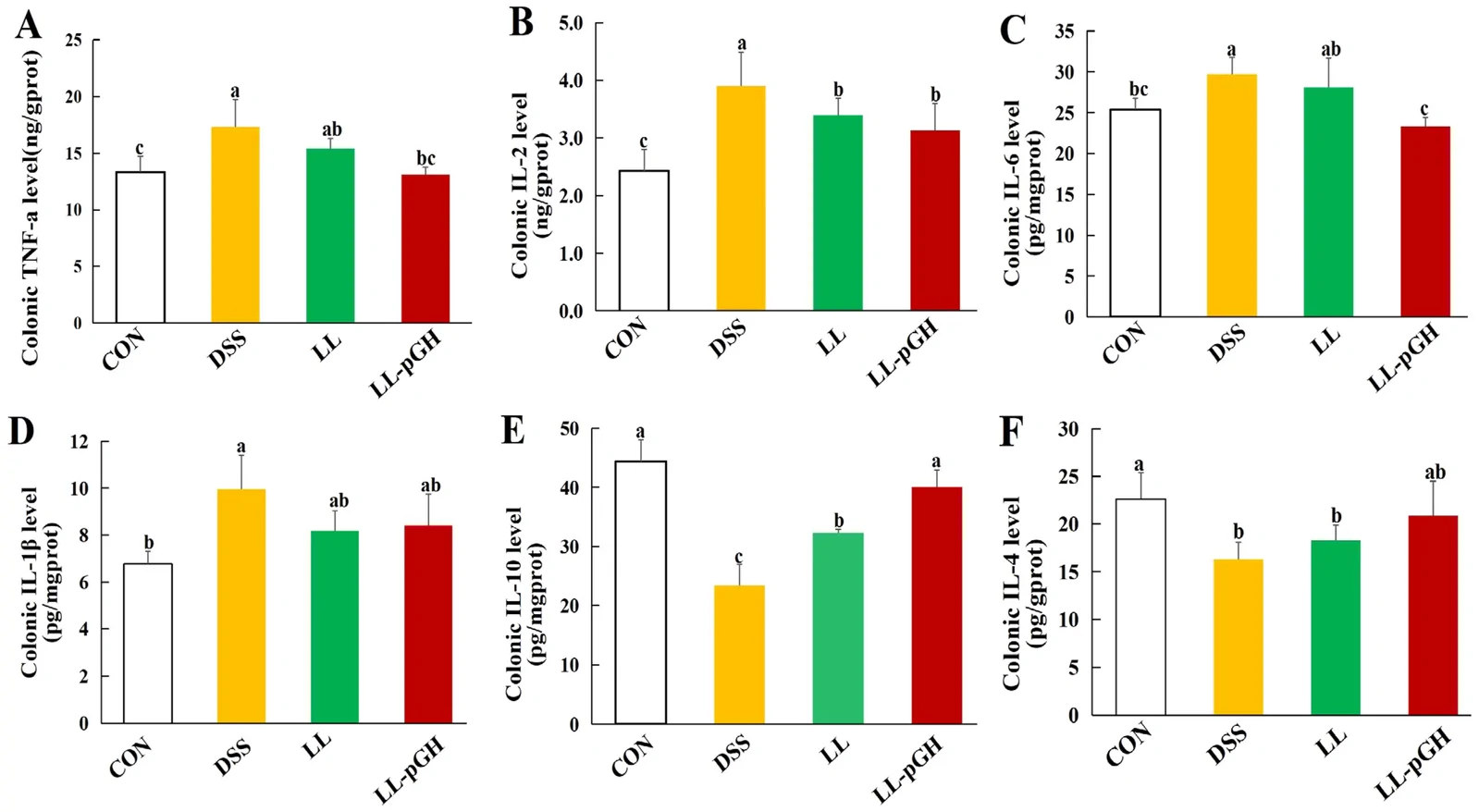
Effect of *L. lactis* (pNZ8148-pGH) on colonic inflammation in DSS-induced colitis. (**A**) Colonic TNF-α level; (**B**) colonic IL-2 level; (**C**) colonic IL-6 level; (**D**) colonic IL-1β level; (**E**) colonic IL-10 level; (**F**) colonic IL-4 level. n = 10 mice per group. Data are expressed as the mean ± SD. Bars without a common letter differ significantly (*p* < 0.05). CON: normal control; DSS: DSS model control; LL: *L. lactis* (pNZ8148); LL-pGH: *L. lactis* (pNZ8148-pGH).

## Data Availability

The original contributions presented in this study are included in the article. Further inquiries can be directed to the corresponding author.
